# Polymorphisms in *EGFR* Gene Predict Clinical Outcome in Unresectable Non-Small Cell Lung Cancer Treated with Radiotherapy and Platinum-Based Chemoradiotherapy

**DOI:** 10.3390/ijms22115605

**Published:** 2021-05-25

**Authors:** Dorota Butkiewicz, Małgorzata Krześniak, Agnieszka Gdowicz-Kłosok, Monika Giglok, Małgorzata Marszałek-Zeńczak, Rafał Suwiński

**Affiliations:** 1 Center for Translational Research and Molecular Biology of Cancer, Maria Skłodowska-Curie National Research Institute of Oncology, Gliwice Branch, 44-102 Gliwice, Poland; Malgorzata.Krzesniak@io.gliwice.pl (M.K.); Agnieszka.Gdowicz-Klosok@io.gliwice.pl (A.G.-K.); 2 II Radiotherapy and Chemotherapy Clinic and Teaching Hospital, Maria Skłodowska-Curie National Research Institute of Oncology, Gliwice Branch, 44-102 Gliwice, Poland; Monika.Giglok@io.gliwice.pl (M.G.); Rafal.Suwinski@io.gliwice.pl (R.S.); 3 Department of Molecular and Systems Biology, Institute of Bioorganic Chemistry, Polish Academy of Sciences, 61-704 Poznań, Poland; mmarszalek@ibch.poznan.pl

**Keywords:** EGFR, polymorphism, lung cancer, radiotherapy, prognosis, therapy outcome, recurrence, radioresponse

## Abstract

For non-small cell lung cancer (NSCLC), radiotherapy (RT) and platinum-based chemotherapy (CHT) are among the main treatment options. On the other hand, radioresistance and cytotoxic drug resistance are common causes of failure. The epidermal growth factor receptor (EGFR) plays an important role in radioresponse and therapy resistance. We hypothesized that single nucleotide polymorphisms (SNPs) in the *EGFR* gene might affect individual sensitivity to these treatments, and thus, therapy outcome and prognosis. The association between functional *EGFR* SNPs and overall (OS), locoregional recurrence-free (LFRS), and metastasis-free (MFS) survival was examined in 436 patients with unresectable NSCLC receiving RT and platinum-based CHTRT. In a multivariate analysis, the rs712830 CC homozygotes showed reduced OS in the whole group (*p* = 0.039) and in the curative treatment subset (*p* = 0.047). The rs712829 TT genotype was strongly associated with decreased LRFS (*p* = 0.006), and the T-C haplotype was a risk factor for locoregional recurrence in our patients (*p* = 0.003). The rs2227983 GG alone and in combination with rs712829 T was an indicator of unfavorable LRFS (*p* = 0.028 and 0.002, respectively). Moreover, significant independent effects of these SNPs on OS, LRFS, and MFS were observed. Our results demonstrate that inherited *EGFR* gene variants may predict clinical outcomes in NSCLC treated with DNA damage-inducing therapy.

## 1. Introduction

Lung cancer has one of the lowest survival rates of all cancer types in the world [1]. Non-small cell lung cancer (NSCLC) accounts for 80–85% of all lung cancer cases, and the majority of patients present at an advanced inoperable stage. In advanced, unresectable NSCLC, radiotherapy (RT), and platinum-based chemotherapy (CHT) still remain the main components of standard care; however, drug and radiation resistance represent serious limitations of these treatments, contributing to recurrence, disease progression, and poor survival [2]. Since such resistance affects a large number of patients, a better understanding of the molecular background of this phenomenon is necessary to identify factors that may help predict treatment outcomes and improve survival.

The epidermal growth factor receptor (EGFR/ErbB-1) is a transmembrane protein belonging to the ErbB family of receptor tyrosine kinases, and its activation triggers diverse signaling pathways essential for the regulation of cell proliferation, differentiation, migration, invasion, and survival [3]. EGFR plays a crucial role in tumorigenesis, and mutations in the *EGFR* gene are used as predictive biomarkers in targeted therapy. Both increased EGFR activation and overexpression, most often resulting from somatic mutations, gene amplification, transcriptional upregulation, or ligand overproduction, promote cancer development and progression, including tumor angiogenesis, metastasis, and resistance to apoptosis [4,5]. EGFR overexpression, frequently observed in NSCLC and other solid tumors, has been associated with disease aggressiveness, higher recurrence rates, and unfavorable prognosis [6,7,8,9,10]. Deregulated EGFR may also contribute to drug and radiation resistance [11,12,13,14].

One of the factors underlying decreased sensitivity to DNA damaging treatment, such as RT or platinum-based CHT, may be enhanced DNA repair. EGFR is known to translocate to the nucleus and participate in DNA damage response (DDR) mechanisms, including DNA damage repair, cell cycle arrest, and apoptosis [14,15]. For example, through direct interaction with the catalytic subunit of DNA-dependent protein kinase (DNA-PKcs), which is a key factor in the non-homologous end joining (NHEJ) repair, EGFR may modulate the removal of DNA lesions induced by ionizing radiation and platinum drugs [16,17]. EGFR has also been found to bind to ERCC1, which is a central component of nucleotide excision repair (NER) machinery essential for the repair of platinum-DNA adducts [18]. Several studies have shown that inhibition of EGFR signaling may increase chemosensitivity and radiosensitivity, thereby improving therapy results [16,19,20,21,22,23,24].

Inherited genetic factors, such as single nucleotide polymorphisms (SNPs), are thought to play a role in the individual response to RT and anticancer drugs, and numerous reports indicate their influence on treatment results and lung cancer prognosis [25,26,27,28]. It is plausible that SNPs altering *EGFR* gene expression, protein levels, or signaling may contribute to variable clinical outcomes and survival in cancer patients. Research on the predictive and prognostic potential of *EGFR* SNPs has mainly focused on EGFR-directed therapy with small-molecule tyrosine kinase inhibitors (TKIs) and anti-EGFR monoclonal antibodies (mAbs). Although the results of these studies are not conclusive, some of them show an association between *EGFR* SNPs and the efficacy of EGFR–targeted treatment in lung cancer as well as in other solid tumors [29,30,31,32,33]. However, very little is known about their possible influence on the effects of RT or CHT with platinum drugs commonly used in NSCLC treatment. Given the role of EGFR in response to radiation and platinum-based CHT, we assumed that *EGFR* gene variants might modulate individual sensitivity to these therapies and patient survival.

Therefore, the purpose of this study was to investigate the potential effect of common *EGFR* gene polymorphisms on clinical outcomes in a relatively large group of 436 patients with unresectable NSCLC receiving RT or platinum-based chemoradiotherapy (CHTRT).

## 2. Results

The median overall survival (OS) in the group was 16.4 months (range 1.2–120.6 months), the median locoregional recurrence-free survival (LRFS) was 17.0 months (range 0.1–108.1 months), and the median metastasis-free survival (MFS) was 25.4 months (range 0–117.1 months). The median follow-up was 58.6 months. During the follow-up period, there were 355 deaths (81%), 161 (37%) patients experienced locoregional recurrence, and 126 (29%) patients developed distant metastasis. The relationship between clinicopathological factors and survival outcomes is presented in Table 1. Advanced clinical stage, poor performance status, smoking, lack of CHT and RT dose < 60 Gy were significantly associated with shorter OS and LRFS, while older age was only associated with inferior OS. In addition, advanced stage and lower RT dose were related to reduced MFS. Minor allele frequencies (MAFs) for all SNPs were consistent with those observed in other European populations [34], and the genotype distributions were in Hardy–Weinberg equilibrium (Table S1). In general, no significant associations between patients’ characteristics and SNPs were observed, with a few exceptions. Namely, there were more rs712830 CC homozygotes in the squamous cell carcinoma (SCC) subgroup compared to the non-SCC subset (70% versus 61%, *p* = 0.044) and the rs712829 T allele was more common in older patients than in younger ones (56% versus 47%, *p* = 0.048) (data not shown).

### 2.1. Individual SNPs and Survival

**Figure 1:**
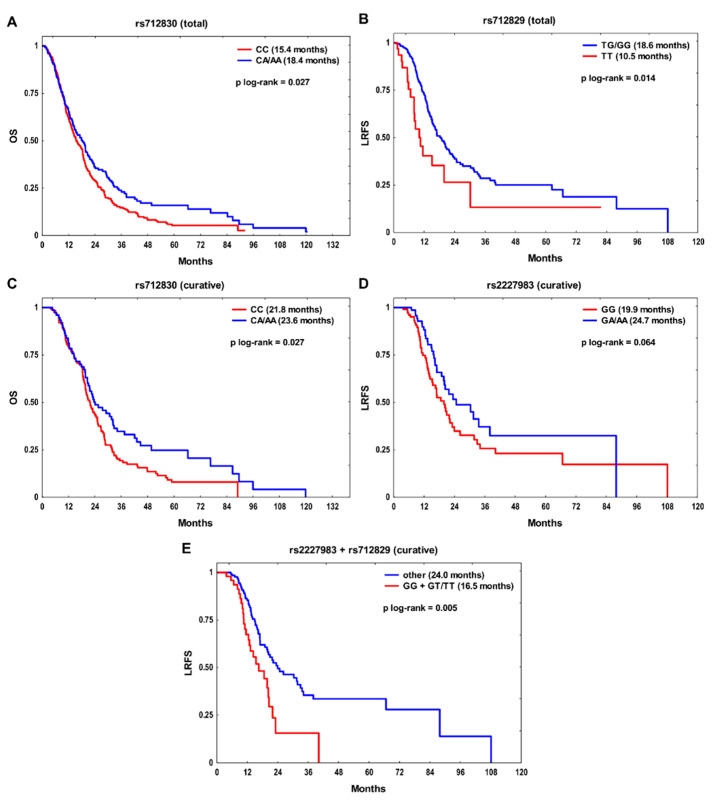
The Kaplan–Meier plots for the effect of (**A**) rs712830 on overall survival (OS) and (**B**) rs712829 on locoregional recurrence-free survival (LRFS) in the whole group, and (**C**) rs712830 on OS, (**D**) rs2227983 on LRFS and (**E**) rs2227983/rs712829 combination on LRFS in the curative treatment subgroup. Median survival time is shown in the brackets.

In the entire study group, the rs712830 CC and rs712829 TT genotypes were significantly associated with poor outcome (Figure 1A,B). The rs712830 CC homozygotes had an increased risk of death (hazard ratio (HR) 1.28, *p* = 0.032 in uni- and HR 1.28, *p* = 0.039 in multivariate models, respectively), while the rs712829 TT homozygotes had a two-fold higher risk of locoregional relapse (*p* = 0.007 in univariate and *p* = 0.006 in multivariate models) (Table 2). In addition, the effect of the examined SNPs on survival was assessed in a more homogeneous subgroup of 205 patients who received radiation therapy or CHTRT with curative intent. In this subset, rs712830 CC was also significantly associated with unfavorable OS in univariate (HR 1.44, *p* = 0.030) and multivariate models (HR 1.44, *p* = 0.047) (Figure 1C, Table 3). The rs2227983 GG genotype was a risk factor for poor LRFS (HR 1.50, *p* = 0.069 in univariate and HR 1.67, *p* = 0.028 in multivariable models) (Figure 1D, Table 3). Patients with rs712829 T allele showed reduced LRFS (HR 1.43, *p* = 0.099 in univariate model) and MFS (HR 1.60, *p* = 0.079 in multivariate model), but both associations were nonsignificant.

Finally, a backward stepwise multiple regression was performed to select independent risk factors for OS, LRFS, and MFS. In the whole group, rs712830 CC, together with clinical factors, was an independent indicator of adverse OS, whereas rs712829 TT had an independent negative effect on LRFS (Table 4). In the curative treatment subgroup, the final model revealed that rs712830 CC was the only independent prognostic factor affecting OS, while the rs2227983 GG genotype, rs712829 T allele, and advanced clinical stage were independent predictors of poor LRFS. The rs712829 T was also an independent risk factor for MFS.

### 2.2. Survival Analysis According to the Combined SNPs

We then decided to construct genotype combinations based on the results presented in Table 2 and Table 3 in order to evaluate the joint effect of several genetic factors on the outcome. Only SNPs with *p* ≤ 0.100 in univariate analysis for a given endpoint were taken into account. Therefore, only one SNP combination could be further assessed, namely rs2227983/rs712829 in relation to LRFS in the curative treatment subset. When all possible genotype combinations were analyzed (i.e., GA/AA + GG, GA/AA + GT/TT, GG + GG, and GG + GT/TT), an interaction was found between rs2227983 and rs712829 in relation to LRFS. Carriers of rs2227983 GG or rs712829 GT/TT had significantly better LRFS than individuals with both adverse genotypes GG + GT/TT (*p* _interaction_ = 0.025, likelihood ratio test). In patients with rs2227983 GG + rs712829 GT/TT an over two-fold increased risk of locoregional recurrence in univariate (HR 2.13, 95% confidence interval (CI) 1.32–3.42, *p* = 0.002) and multivariate analysis (HR 2.21, 95% CI 1.34–3.65, *p* = 0.002) was observed compared to the carriers of other genotype combinations (Figure 1E). Moreover, the rs2227983 GG + rs712829 GT/TT combination was an independent predictor of unfavorable LRFS in the curative treatment subgroup (Table 4).

### 2.3. Haplotype Analysis

**Figure 2:**
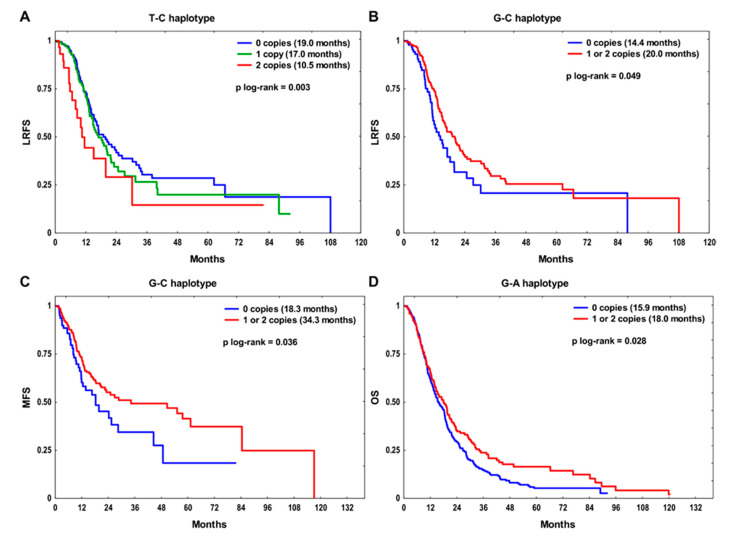
The Kaplan–Meier survival analysis according to the rs712829 and rs712830 haplotypes: (**A**) T-C haplotype and (**B**) G-C haplotype in relation to locoregional recurrence-free survival (LRFS), (**C**) G-C haplotype in relation to metastasis-free survival (MFS), and (**D**) G-A haplotype in relation to overall survival (OS). Median survival time is shown in the brackets.

Linkage disequilibrium (LD) analysis revealed that there were two SNPs in LD—*EGFR* rs712829 and rs712830 (*D’* = 0.886, *r*^2^ = 0.079, *p* < 1 × 10^−5^) (Figure S1). Therefore, four rs712829-rs712830 haplotypes were defined with the following frequencies: 51.7% for G-C, 29.0% for T-C, 18.6% for G-A, and 0.7% for T-A. Survival analysis for each endpoint was performed only for haplotypes with a frequency above 1%. The T-C haplotype was significantly associated with poor LRFS in our dataset (*p* = 0.003, Figure 2A). The T-C/T-C diplotype carriers were at the increased risk of locoregional recurrence in univariate (HR 1.92, 95% CI 1.12–3.30, *p* = 0.019) and multivariate models (HR 1.76, 95% CI 1.02–3.05, *p* = 0.043), compared to the T-C non-carriers. The G-C haplotype showed protective effect on LRFS (*p* = 0.049) and MFS (*p* = 0.036) (Figure 2B,C). Patients with at least one copy of G-C haplotype had a significantly lower risk of locoregional (HR 0.69, 95% CI 0.49–0.99, *p* = 0.041 in univariate analysis) and distant relapse (HR 0.66, 95% CI 0.45–0.97, *p* = 0.033 in univariate, and HR 0.57, 95% CI 0.38–0.86, *p* = 0.007 in multivariate models) than carriers of zero copies. Finally, the G-A haplotype was found to be protective with respect to OS (*p* = 0.028) (Figure 2D). The G-A carriers showed reduced risk of death compared to non-carriers (HR 0.78, 95% CI 0.62–0.98, *p* = 0.032 in univariate, and HR 0.80, 95% CI 0.63–1.01, *p* = 0.057 in multivariate analysis).

## 3. Discussion

In this exploratory study, we focused on functional SNPs in the *EGFR* gene—two located in the regulatory region and one in the coding region—and examined the association of these SNPs, their combinations, as well as haplotypes with three survival endpoints in NSCLC patients receiving RT and platinum-based CHTRT. We found that rs712830 CC, rs712829 TT or GT/TT, and rs2227983 GG genotypes were significant risk factors for unfavorable outcomes in our cohort.

Furthermore, in the final multivariate analysis that incorporated clinicopathological factors, these genotypes were identified as independent genetic predictors of poor OS, LRFS, and MFS.

Literature data on the relationship between *EGFR* SNPs and clinical outcomes in solid tumors treated with RT and conventional, non-targeted anticancer drugs such as platinum compounds are very limited. In lung cancer, the analyzed SNPs were previously investigated mainly in the context of response to targeted treatment with EGFR TKIs or, in colorectal and head and neck cancer, to treatment with anti-EGFR mAbs such as cetuximab [29,30,31,32]. The vast majority of these studies involved small groups of patients. In a very recent meta-analysis, Jurisic et al. [33] summarized the results in NSCLC, showing that rs712829 significantly affected OS and PFS in patients receiving gefitinib or erlotinib. In turn, the largest study among lung cancer patients given RT and standard CHT was carried out in a Chinese population; however, the authors found no relationship between the SNPs we examined and prognosis [35]. In our NSCLC group, the rs712829 variant was independently associated with locoregional and distant relapse, while rs712830 CC correlated with worse OS in the whole group as well as in the more homogeneous curative treatment subgroup. This corresponds to the results reported by Guo et al. [36], who found the rs712829 T allele to be a risk factor for pleural metastasis in lung adenocarcinoma and observed increased EGFR protein expression in tumor tissues with T variant. The rs712830 CC genotype was also identified as a negative prognostic factor in glioblastoma [37]. Conversely, in two other small studies, a better response to chemoradiation was associated with rs712829 T in rectal cancer Danish patients [38], while rs712830 A correlated with reduced progression-free survival and higher cancer risk in a lung adenocarcinoma group from India [39]. Both SNPs, rs712829 (–216G > T) and rs712830 (–191C > A), are located in multiple transcriptional start site regions of the gene promoter and may influence *EGFR* regulation. The –216G > T is the Sp1 binding site polymorphism, and –191C > A is situated in close proximity to one of the transcription initiation sites. The rs712829 T variant has been demonstrated to increase promoter activity by 30%, whereas the rs712829/rs712830 T-C haplotype was associated with 40% higher mRNA expression in vivo, as compared to G-C [40]. This is consistent with the results of our haplotype analysis showing that the T-C haplotype was a risk factor for recurrence, and G-C and G-A haplotypes were protective for the outcome in our NSCLC cohort. Since increased EGFR expression has been found to correlate with lower sensitivity to RT and platinum drugs, and poor survival [6,8,10,12,13], it is possible that these two variants, separately and in combination, affect individual response to these treatments by modulating gene expression and possibly protein levels, and, consequently, lung cancer prognosis. Furthermore, in the study by Liu et al. [41], the –216T allele frequency was found to be significantly higher in NSCLC patients with *EGFR* tyrosine kinase domain mutations, suggesting that rs712829 SNP may contribute to the development of these mutations, in particular activating deletions in exon 19, and thus leading to a more invasive phenotype.

Here, we also found the rs2227983 GG genotype, alone and combined with rs712829 T variant, conferred an increased risk of locoregional recurrence in patients treated with the curative intent. This effect was stronger for the combination than for individual SNPs, highlighting the usefulness of a panel of variants in risk assessment. Both rs2227983 GG and rs2227983 GG + rs712829 T proved to be independent risk factors in these patients. Non-synonymous rs2227983 SNP (also referred to as R497K or rs11543848) causes a G to A substitution leading to Arg (R) to Lys (K) change in codon 521 in exon 13, and is located in the extracellular domain within subdomain IV of EGFR. The R521K has been shown to reduce ligand binding, tyrosine kinase activation, growth signals stimulation, and induction of proto-oncogenes [42]. Therefore, one may assume that GG (R) genotype is associated with higher EGFR activity, increased signaling, and possible lower effectiveness of cytotoxic treatment, which would be in line with our observations. This is also in agreement with the results of a Chinese study involving lung adenocarcinoma, in which the 521R variant was related to higher protein levels in tumors and increased metastatic risk [36]. In a small NSCLC subgroup from Japan treated with surgery and platinum-based CHT, rs2227983 GG homozygotes had a worse prognosis than A allele carriers [43]. Similarly, cervical cancer patients with GG showed lower sensitivity to radiochemotherapy and a higher risk of recurrence or metastasis [44]. In locally advanced pharyngolaryngeal cancer treated with cisplatin-based CHTRT, the G variant was associated with poor five-year OS [45]. In metastatic colorectal cancer, GG homozygotes had a lower response to oxaliplatin-based CHT and unfavorable survival [46]. The 521R variant has been shown to correlate with poor prognosis in bladder cancer patients and in men with colon cancer [47,48]. The independent effects of rs2227983 on the risk of recurrence as well as rs712829 on the risk of metastasis observed in our curative treatment subset, in which almost 90% of patients received platinum-based doublet, may indicate a possible role of the above variants in the molecular mechanisms of chemoresistance to these drugs.

Taken together, our data suggest that in NSCLC patients undergoing RT and CHTRT with platinum compounds, the rs712829 T variant and the rs2227983 GG genotype promote cancer relapse, while the rs712830 CC genotype increases the risk of death. Therefore, our results support the hypothesis that common inherited *EGFR* gene variants associated with elevated gene expression and protein activity may predict poorer efficiency of these therapies and clinical course of disease. To the best of our knowledge, this is the first study investigating the prognostic value of *EGFR* SNPs in the Caucasian population of NSCLC patients in the context of treatment with RT and platinum drugs. Our data could help to better understand the contribution of host genetic profile to individual treatment sensitivity and lung cancer progression, which may lead to the development of personalized therapeutic strategies to improve local recurrence, metastasis rate, and survival.

There are some limitations in our study that should be taken into consideration. Despite ethnic homogeneity and a long follow-up period, the medium size of our patient group may have an impact on the reliability of results. Nevertheless, it is one of the largest NSCLC cohorts to date, in which the prognostic value of *EGFR* SNPs has been evaluated. Moreover, our observations concern the Polish Caucasian population and may not translate to other populations. Due to the scarce literature data on *EGFR* SNPs in RT- and CHT-treated cancer patients, we were unable to fully relate our findings to the observations of other authors, which may have affected the interpretation of the results. Thus, as our study is exploratory in nature, additional research is necessary to elucidate the role of *EGFR* SNPs in modulating the sensitivity to RT and platinum drugs and lung cancer prognosis. Another limitation is the widespread introduction of new diagnostic tools and new therapies that were largely not available at the time of this study. This includes tumor gene sequencing as well as the use of specific targeted drugs for tumors with targetable alterations (e.g., EGFR, ALK, ROS1, NTRK, BRAF, or HER inhibitors). The same refers to the increasingly common utility of immunotherapy alone or combined with cytotoxic therapies. One may postulate that the identification of *EGFR* polymorphisms may in the future contribute to a better prediction of the benefits from a given therapy and delivery of optimized treatment strategies.

## 4. Materials and Methods

### 4.1. Patients and Treatment

The study group included 436 Caucasian patients with unresectable NSCLC who received first-line treatment between October 2006 and February 2015. The demographic and clinical data are summarized in Table 1. The median age of the group was 63 years (mean age ± standard deviation was 63.1 ± 9.1 years). The majority of patients were males (71%) and smokers (94%). Squamous cell carcinoma (SCC) was diagnosed in 266 (61%) individuals. Out of all patients, 398 (91%) had clinical stage III or IV. The patients in clinical stages I and II were not referred for surgery due to comorbidities. All patients were treated with RT with a total dose ≥20 Gy, and 301 (69%) received 1–6 courses of platinum-based doublet (cisplatin or carboplatin with vinorelbine, pemetrexed, etoposide, or gemcitabine). Thoracic RT with curative intent (≥60 Gy) was administered to 205 (47%) patients. Most of these patients received 2–4 courses of induction chemotherapy followed by linear accelerator-based radiation therapy with PET/CT 3D treatment planning and image-guided treatment delivery. The patients in stages I and II received gated stereotactic radiotherapy alone.

### 4.2. SNP Identification

The analyzed SNPs included *EGFR* rs712829, rs712830, and rs2227983. Based on a literature search, we selected SNPs with well-documented functional significance that showed prognostic/predictive effect in solid tumors treated with non-targeted therapy, and their minor allele frequency in the European Caucasian population was >15% [34]. DNA was isolated from frozen peripheral blood with a Genomic Maxi AX kit (A&A Biotechnology, Gdynia, Poland). The rs2227983 genotypes were determined using TaqMan^®^ SNP Genotyping Assay C_16170352_20 (Applied Biosystems, Foster City, CA, USA), according to the manufacturer’s standard protocol. The polymerase chain reaction-restriction fragment length polymorphism (PCR-RFLP) method was used to identify rs712829 and rs712830 SNPs. The forward primer 5′-CTG CTC CTC CCG ATC CCT CCT CCG CGG C-3′ and reverse primer 5′-GAG GTG GCC TGT CGT CCG GTC T-3′ were used for amplification. The reaction was performed in 25 µL total volume containing 50 ng of genomic DNA, 0.2 mM of each dNTP (Fermentas, Vilnius, Lithuania), 12.5 pmol of each primer (BioTeZ, Berlin-Buch, Germany), 1x PCR buffer, 1.5 mM MgCl_2_, 0.6 U of HotStar Taq DNA polymerase (Qiagen, Hilden, Germany) and 5% dimethyl sulfoxide. The initial denaturation at 95 °C for 15 min was followed by 35 cycles of denaturation at 95 °C for 30 s, annealing at 65 °C for 30 s and elongation at 72 °C for 30 s, ending with 72 °C for 7 min. Then, PCR products (8 µL) were separately digested overnight with 7 U of *Bse*RI (for rs712829) or *Sac*II (for rs712830) restriction enzymes (New England BioLabs, Ipswich, MA, USA), and the fragments were separated on 3–4% ethidium bromide-stained agarose gels. Genotyping was repeated in 50 randomly selected samples, and 100% concordance was found.

### 4.3. Statistical Analysis

Clinical endpoints of the study were overall survival (OS), locoregional recurrence-free survival (LRFS), and metastasis-free survival (MFS). OS was defined as the interval between diagnosis and death from any cause or the last known date alive (censored data). LRFS and MFS were calculated from the date of treatment initiation to the date of documented progression of the primary tumor or locoregional lymph nodes (for LRFS) or the date of distant relapse (for MFS). The cases that did not locally progress or did not have distant metastases were censored at the date of the last follow-up. The association between each SNP and the outcome was tested under additive, dominant and recessive genetic models, and the best model (i.e., the one with the lowest *p*-value) was selected for the final analysis. Survival curves were determined with the Kaplan–Meier method and compared by log-rank test. The hazard ratios (HRs) with 95% confidence intervals (CIs) were estimated using univariate and multivariate Cox proportional hazards regression. Multivariate models were adjusted for age at diagnosis, sex, histology, clinical stage, performance status, smoking status, CHT use, and radiation dose. A backward stepwise regression procedure was also used. Pearson’s chi-square test was used to assess differences between variables and test for deviation from the Hardy–Weinberg equilibrium. Spearman’s correlation was also applied. Haplotype blocks were determined according to Gabriel et al. [49] in Haplowiev 4.2 [50,51]. The degree of linkage disequilibrium (LD) between SNPs was examined using *D’* and *r*^2^. Haplotypes and their frequencies were estimated using PHASE v2.1.1. [52]. Two-sided *p*-values ≤ 0.05 were considered to be statistically significant. Statistica 13.1 (TIBCO Software Inc., Palo Alto, CA, USA) and R statistical package v3.3.1. (The R Foundation for Statistical Computing, Vienna, Austria, https://www.r-project.org, accessed on 1 October 2020) were used for calculations.

## 5. Conclusions

The presented data show that functional *EGFR* genetic variants, such as rs712829, rs712830, and rs2227983, may significantly modify the clinical outcomes in patients with inoperable NSCLC receiving DNA damage-inducing anticancer therapy, such as RT and platinum-based CHTRT. The *EGFR* SNPs may act as independent molecular prognostic predictors in these patients; however, larger-scale studies are warranted to confirm our observations.

## Figures and Tables

**Table 1:** Patient characteristics and survival.

Feature		OS				LRFS				MFS			
	*n* (%)	Event	mOS	HR (95% CI)	*p*-Value	Event	mLRFS	HR (95% CI)	*p*-Value	Event	mMFS	HR (95% CI)	*p*-Value
		*n*				*n*				*n*			
Sex													
Female	125 (29)	100	18.4	1		54	19.8	1		45	20.8	1	
Male	311 (71)	255	15.4	1.23 (0.97–1.55)	0.087	107	17	1.04 (0.75–1.45)	0.796	81	26.9	0.94 (0.65–1.35)	0.726
*p* log-rank			0.077				0.786				0.713		
Age													
<63	206 (47)	164	19.5	1		84	21	1		78	22.5	1	
≥63	230 (53)	191	14	1.38 (1.12–1.71)	0.002	77	14.1	1.36 (0.99–1.85)	0.055	48	44.5	0.82 (0.57–1.17)	0.273
*p* log-rank			0.002				0.058				0.276		
Histology													
SCC	266 (61)	226	18.4	1		105	16.3	1		72	25.4	1	
AC	77 (18)	62	17.3	0.85 (0.64–1.13)	0.258	21	31.8	0.57 (0.36–0.91)	0.019	23	26.9	1.18 (0.73–1.89)	0.5
NOS	93 (21)	67	18	0.77 (0.59–1.02)	0.064	35	16.5	0.95 (0.64–1.39)	0.777	31	18.3	1.23 (0.80–1.87)	0.345
*p* log-rank			0.433				0.318				0.478		
Clinical stage													
I–II	38 (9)	31	23.6	1		10	24	1		9	50.6	1	
III	317 (73)	255	18.1	1.24 (0.86–1.81)	0.253	127	16.9	2.15 (1.13–4.11)	0.02	102	25.4	1.66 (0.84–3.29)	0.144
IV	81 (18)	69	10.4	2.29 (1.49–3.50)	1 × 10^−4^	24	13.2	3.53 (1.68–7.42)	9 × 10^−4^	15	4.2	11.85 (5.15–27.30)	<1 × 10^−6^
*p* log-rank			<1 × 10^−5^				8 × 10^−4^				3 × 10^−4^		
ECOG/Zubrod PS													
0	114 (26)	93	20	1		49	20.4	1		39	44.5	1	
1	290 (67)	235	15.2	1.34 (1.05–1.71)	0.017	101	16.5	1.33 (0.94–1.89)	0.102	82	25	1.33 (0.91–1.96)	0.145
2	32 (7)	27	11.2	2.29 (1.49–3.53)	2 × 10^−4^	11	7.8	3.19 (1.64–6.18)	6 × 10^−4^	5	16.1	1.64 (0.64–4.18)	0.303
*p* log-rank			1 × 10^−4^				3 × 10^−4^				0.268		
Smoking status													
Never	25 (6)	17	26.4	1		10	33.6	1		9	–	1	
Ever	411 (94)	338	15.5	1.98 (1.22–3.23)	0.006	151	16.5	1.82 (0.96–3.47)	0.068	117	25.4	1.24 (0.63–2.44)	0.542
*p* log-rank			6 × 10^−4^				0.014				0.458		
Chemotherapy													
No	135 (31)	111	9.8	1		39	13.1	1		22	28.5	1	
Yes	301 (69)	244	19.4	0.50 (0.40–0.63)	<1 × 10^−6^	122	19.8	0.57 (0.40–0.82)	0.002	104	25	0.89 (0.56–1.41)	0.61
*p* log-rank			<1 × 10^−5^				0.016				0.686		
Radiation dose													
<60 Gy	231 (53)	192	11.4	1		71	13.1	1		58	12.1	1	
≥60 Gy	205 (47)	163	22.5	0.45 (0.37–0.56)	<1 × 10^−6^	90	21	0.47 (0.34–0.64)	3x10^−6^	68	48.7	0.40 (0.28–0.57)	<1 × 10^−6^
*p* log-rank			<1 × 10^−5^				2 × 10^−5^				1 × 10^−5^		

OS: overall survival; LRFS; locoregional recurrence-free survival; MFS: metastasis-free survival; mOS: median OS (months); mLRFS: median LRFS (months); mMFS: median MFS (months); HR: hazard ratio; CI: confidence interval; SCC: squamous cell carcinoma; AC: adenocarcinoma; NOS: not otherwise specified; ECOG: Eastern Cooperative Oncology Group; PS: performance status.

**Table 2:** Survival analysis according to individual SNPs in the whole group.

Endpoint	SNP	Genotype	Event/*n*	uHR (95% CI)	*p*-Value	mHR (95% CI)	*p*-Value
OS	rs2227983	GA/AA	157/196	1		1	
		GG	190/231	1.02 (0.82–1.26)	0.878	1.04 (0.84–1.29)	0.708
	rs712830	CA/AA	119/147	1		1	
		CC	236/289	1.28 (1.02–1.60)	**0.032**	1.28 (1.01–1.61)	**0.039**
	rs712829	GG	177/211	1		1	
		GT/TT	178/225	1.08 (0.87–1.33)	0.478	0.96 (0.78–1.19)	0.723
LRFS	rs2227983	GA/AA	69/196	1		1	
		GG	87/231	1.13 (0.83–1.56)	0.44	1.25 (0.91–1.73)	0.172
	rs712830	CA/AA	57/147	1		1	
		CC	104/289	1.11 (0.80–1.55)	0.516	1.02 (0.73–1.44)	0.905
	rs712829	TG/GG	143/402	1		1	
		TT	18/34	1.97 (1.21–3.23)	**0.007**	2.03 (1.23–3.34)	**0.006**
MFS	rs2227983	GA/AA	58/196	1		1	
		GG	66/231	0.91 (0.64–1.30)	0.616	0.89 (0.62–1.28)	0.532
	rs712830	CA/AA	44/147	1		1	
		CC	82/289	1.24 (0.86–1.80)	0.252	1.31 (0.89–1.94)	0.171
	rs712829	GG	62/211	1		1	
		GT/TT	64/225	1.17 (0.82–1.67)	0.377	1.29 (0.88–1.87)	0.188

OS: overall survival; LRFS: locoregional recurrence-free survival; MFS: metastasis-free survival; SNP: single nucleotide polymorphism; uHR: univariate hazard ratio; mHR: multivariate hazard ratio; CI: confidence interval; *p* ≤ 0.05 shown in bold.

**Table 3:** Survival analysis according to individual SNPs in the subset treated with curative intent.

Endpoint	SNP	Genotype	Event/*n*	uHR (95% CI)	*p*-Value	mHR (95% CI)	*p*-Value
OS	rs2227983	GA/AA	70/91	1		1	
		GG	87/107	1.01 (0.73–1.38)	0.97	1.03 (0.74–1.43)	0.878
	rs712830	CA/AA	57/77	1		1	
		CC	106/128	1.44 (1.04–2.01)	**0.03**	1.44 (1.05–2.07)	**0.047**
	rs712829	GG	81/102	1		1	
		GT/TT	82/103	1.28 (0.94–1.75)	0.12	1.20 (0.86–1.67)	0.273
LRFS	rs2227983	GA/AA	33/91	1		1	
		GG	53/107	1.50 (0.97–2.32)	0.069	1.67 (1.06–2.64)	**0.028**
	rs712830	CA/AA	36/77	1		1	
		CC	54/128	1.14 (0.74–1.76)	0.54	1.00 (0.61–1.64)	0.991
	rs712829	GG	43/102	1		1	
		GT/TT	47/103	1.43 (0.94–2.17)	0.099	1.32 (0.84–2.07)	0.23
MFS	rs2227983	GA/AA	30/91	1		1	
		GG	37/107	0.91 (0.56–1.49)	0.722	0.99 (0.59–1.64)	0.954
	rs712830	CA/AA	24/77	1		1	
		CC	44/128	1.50 (0.90–2.50)	0.115	1.45 (0.85–2.50)	0.174
	rs712829	GG	34/102	1		1	
		GT/TT	34/103	1.44 (0.88–2.37)	0.145	1.60 (0.95–2.71)	0.079

OS: overall survival; LRFS: locoregional recurrence-free survival; MFS: metastasis-free survival; SNP: single nucleotide polymorphism; uHR: univariate hazard ratio; mHR: multivariate hazard ratio; CI: confidence interval; *p* ≤ 0.05 shown in bold.

**Table 4:** Independent risk factors—individual SNPs and tested combinations.

Total			
**Endpoint**	**Variable**	**HR (95% CI)**	***p*** **-Value**
OS	rs712830 CC	1.31 (1.04–1.64)	0.02
	Stage III	1.59 (1.07–2.36)	0.02
	Stage IV	1.93 (1.22–3.06)	0.005
	Ever smoking: yes	2.02 (1.24–3.30)	0.005
	Chemotherapy: no	1.84 (1.43–2.37)	2 × 10^−6^
	RT dose < 60 Gy	1.74 (1.36–2.23)	1.2 × 10^−5^
LRFS	rs712829 TT	2.15 (1.31–3.52)	0.003
	SCC	1.40 (1.01–1.96)	0.046
	Zubrod PS 2	2.08 (1.10–3.93)	0.025
	Stage III	2.61 (1.32–5.14)	0.006
	Stage IV	3.11 (1.39–7.00)	0.006
	Chemotherapy: no	1.77 (1.18–2.66)	0.006
	RT dose < 60 Gy	1.70 (1.17–2.48)	0.006
MFS	Stage IV	4.75 (2.62–8.59)	<1 × 10^−6^
	RT dose < 60 Gy	2.01 (1.36–2.97)	0.0004
**Curative treatment subgroup**			
**Endpoint**	**Variable**	**HR (95% CI)**	***p*** **-value**
OS	rs712830 CC	1.44 (1.04–2.01)	0.03
LRFS	rs2227983 GG	1.57 (1.01–2.43)	0.046
	rs712829 GT/TT	1.66 (1.07–2.57)	0.023
	Stage III–IV	2.39 (1.10–5.19)	0.028
LRFS	rs2227983 GG + rs712829 GT/TT	2.00 (1.25–3.22)	0.004
	Stage III–IV	2.24 (1.03–4.88)	0.041
MFS	rs712829 GT/TT	1.68 (1.01–2.78)	0.045
	Age < 63 years	1.71 (1.00–2.93)	0.049
	Non–SCC	1.73 (1.06–2.82)	0.028

OS: overall survival; LRFS: locoregional recurrence-free survival; MFS: metastasis-free survival; HR: hazard ratio; CI: confidence interval; SCC: squamous cell carcinoma; PS: performance status; RT: radiotherapy.

## Data Availability

The data presented in this study are available on request from the corresponding author.

## Supplementary Materials

The following are available online at https://www.mdpi.com/article/10.3390/ijms22115605/s1.
