# Supplementary material for: Polymorphisms in EGFR Gene Predict Clinical Outcome in Unresectable Non-Small Cell Lung Cancer Treated with Radiotherapy and Platinum-Based Chemoradiotherapy
Source: Int J Mol Sci. 2021 May 25;22(11):5605. doi: 10.3390/ijms22115605 (PMC8197839; doi:10.3390/ijms22115605)

Table S1. The genotype distribution in 436 NSCLC patients.

SNP	Genotype	n (%)	Allele	Function class [a]	Regulome DB rank [b]	MAF
rs2227983	GG	231 (53)	Arg521Lys	NS; splicing	5	0.26
	GA	171 (39)	G>A	(ESE/ESS)		
	AA	25 (6)				
	Missing	9 (2)				
rs712830	CC	289 (66)	–191 C>A	promoter; TFBS;	4	0.19
	CA	126 (29)		splicing (ESE/ESS)		
	AA	21 (5)				
rs712829	GG	211 (48)	–216 G>T	promoter; TFBS;	2a	0.30
	GT	191 (44)		splicing (ESE/ESS)		
	TT	34 (8)				

NSCLC, non–small cell lung cancer; SNP, single nucleotide polymorphism; MAF, minor allele frequency; NS, nonsynonymous; TFBS, transcription factor binding site; ESE/ESS; exonic splicing enhancer/exonic splicing silencer; [a] https://www.regulomedb.org ; [b] https://snpinfo.niehs.nih.gov/snpinfo/snpfunc.html

Figure S1. Linkage disequilibrium (LD) analysis and haplotype block structure for three *EGFR* polymorphisms examined in the study—rs712830 (EGFR –216), rs712829 (EGFR –191) and rs2227983 (EGFR 521). The numbers represent the pairwise *D′* values expressed as percentages. The darkness of the cells indicates the strength of LD.

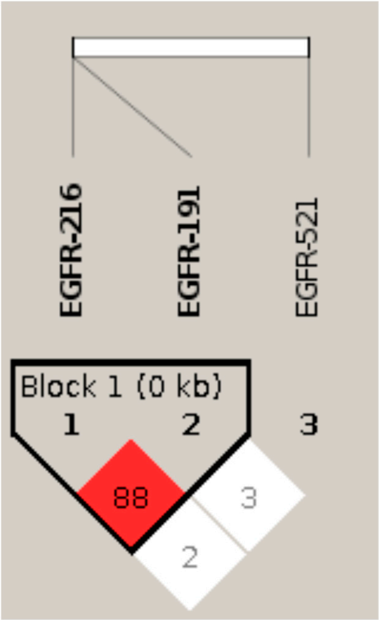

Supplement: Supplementary file 1 [file ijms-22-05605-s001.zip › ijms-1197612-supplementary/EGFR snps in NSLC Supplementary Materials tables figures MDPI style.pdf]
